# Which Tools in Multimedia Are Best for Learning Outcomes? A Study Grounded in Cognitive Load Structures

**DOI:** 10.3389/fpsyg.2021.545335

**Published:** 2021-07-02

**Authors:** Glenn-Egil Torgersen, Ole Boe

**Affiliations:** ^1^Center for Security, Crisis Management and Emergency Preparedness, School of Business, University of South-Eastern Norway, Horten, Norway; ^2^Department of Industrial Economics, Strategy and Political Science, USN School of Business, University College of Southeast Norway, Drammen, Norway

**Keywords:** compositions/tools in multimedia, learning outcome, multimedia learning, working memory, cognitive load theory, dual-coding theory, human–computer interaction, crisis communication

## Abstract

The main objective of this study is to investigate the importance of three compositions in multimedia for learning outcomes (LOs) in relation to individual differences in short-term memory (STM) capacity. The study is based on a survey of 378 individuals at the bachelor level (military officers, teachers, and psychology students). The LOs of three different multimedia compositions (means) were tested. This applied to individuals with low, medium, and high STM capacity. The results show that the successive presentation (Type II) of learning materials through multiple representation forms/channels (speech, pictures, and screen text/labels) provides a better LO than just speech (Type I) and simultaneous presentation (Type III). Overall, visual and verbal channel capacities did not contribute to the LO in any of the three tools tested, but some specific STM capacity types or substructures (visual and verbal progressive capacities) and non-verbal (RAPM) types have significance, particularly in exploiting successive presentation (Type II) for learning. Although the tools used in the multimedia educational material had a low cognitive load, the individuals with low capacity learned relatively less than the individuals with higher capacity. A symbolic form of expression was introduced concerning the relationship between cognitive load structure (CLS) and LOs through various tools in multimedia as an aid in the theoretical and empirical analyses. This is referred to as the *CLS-LO formula*. The main assumption of this study, based on previous empirical and theoretical ones, is that the relationship between CLS and LO is expressed with the following CLS-LO formula: ${\mathrm{CLS}}_{\mathrm{Type}\;\mathrm{III}}>{\mathrm{CLS}}_{\mathrm{Type}\;\mathrm{II}}>{\mathrm{CLS}}_{\mathrm{Type}\;I}\to {\mathrm{LO}}_{\mathrm{Type}\;\mathrm{III}}>{\mathrm{LO}}_{\mathrm{Type}\;I}>{\mathrm{LO}}_{\mathrm{Type}\;\mathrm{II}}$. Based on this study, the relationship became: ${\mathrm{CLS}}_{\mathrm{Type}\;\mathrm{III}}>{\mathrm{CLS}}_{\mathrm{Type}\;I}>{\mathrm{CLS}}_{\mathrm{Type}\;\mathrm{II}}\to {\mathrm{LO}}_{\mathrm{Type}\;\mathrm{II}}>{\mathrm{LO}}_{\mathrm{Type}\;I}={\mathrm{LO}}_{\mathrm{Type}\;\mathrm{III}}$. This basic research study is primarily a contribution to understanding underlying cognitive processes in STM and their importance for learning in multimodal forms compared with analogue text. The findings will also be relevant as a basis for performance analysis and decision-making under high information pressure, risk, and unpredictable conditions.

## Introduction

There is no doubt that studies based on Cognitive Load Theory (CLT) and capacity studies on working memory or short-term memory (STM) have had an impact on the organization of learning and teaching in schools and education (Sweller, 1999; Mayer, 2014; Paas and Ayres, 2014; Sweller et al., 2019). Indeed, human cognitive limitations cannot be ignored, concerning operators and decision-makers in digitally heavy work environments, such as control rooms in the oil sector, transport sector, or cyber defense, where information pressure and risk are simultaneously high. Therefore, the capacity of working memory is a key assessment factor in research on competence in digital work and learning environments.

An operator working on human-computer interaction can quickly experience that his or her mental resources can be overloaded, thus leading to impairment in cognitive functioning (Oviatt, 2006). In addition, looking at the design and execution of cybersecurity programs, it becomes evident that the extent of human resilience when dealing with cognitive load and demands has been insufficiently studied (Dreibelbis et al., 2018). Linked with this is an increasing demand for qualified cyber personnel due to the increased utility of and reliance upon cyberspace in military operations (Champion et al., 2014), which leads to a necessity to investigate the relevant cognitive load factors for these types of personnel. As stated by Jøsok (2020), the available research literature confirms that cyber operators are subject to high cognitive load. This is due to the information-intensive character of work, such as network surveillance (D’Amico et al., 2005), organizational factors of a network-enabled operations environment (Buchler et al., 2016), and the need to perform low-level analysis and high-level analysis continuously (McClain et al., 2015).

This also applies to situations where multimedia is used as an educational aid, but there are still many unanswered scientific questions, not least regarding the importance of STM capacity for learning outcomes (LOs) where various multimedia tools are involved.

Moreover, recent critical review studies (Anmarkrud et al., 2019; Rey et al., 2019) on multimedia-based learning in light of CLT have shown that many previous studies have not measured STM capacity for the same respondents who are part of experiments that investigate LOs from multimedia. LO results have instead been assessed in relation to theoretical models of STM or other independent studies concerning STM capacity that have been conducted with other respondents under conditions completely different from those in the measurement of LOs. In addition, unvarnished STM tests have often been used, which do not specify different subtypes of channel capacity compared with LOs from learning sources with different characteristics, tools, and information load.

Cognitive Load Theory-based review studies specifically emphasize the importance of the environment for performance measurement of both learning and STM capacity (Choi et al., 2014). Controllable laboratory conditions can produce results that experiments conducted in realistic environments, such as classrooms and activities similar to regular teaching, cannot, and few CLT-based studies have been conducted under such conditions (Cowan, 2014).

Therefore the main objective of this study is to investigate the importance of three instruments in multimedia for LOs in relation to individual differences in STM capacity. Both LOs’ STM capacity was measured on the same respondents in the same experiment and under the same conditions, as well as in environments that may be similar to a normal teaching situation in school and education. LOs were measured with a knowledge test (Table 1) after a presentation consisting of digital information and analogue text, whereas levels of STM capacity, such as substructures of visual and verbal channel structures were measured with a digital test (see Tables 2 and 3). The overarching purpose of the study is, thus, to be of contribution to a new understanding of the importance of STM in learning with multimedia.

**Table 1:** Overview of the questions used to measure learning outcomes for the three types of instruments.

Theme questions for instrument type (information sequences)	Content of the instrument/Cognitive load structure (CLS-LO formula)
**Type I questions**	
-*When the western farmers met at the court instance (D)*.-*Took initiative for regional land meetings (D)*	*Real comment*: a commentator is shown (live) while he or she tells a professional content (AVS). Background has little or no direct connection with the specific professional message
-*The national collection process (D)*	$\left({\mathrm{VU}}_{\mathrm{unrel}}\right).$
-*Date of Knut the mighty (D)*	
-*The king and the church’s need for regional meeting places (CU)*	${\mathrm{CLS}}_{\mathrm{Type}\;I}=\mathrm{AVS}+{\mathrm{VI}}_{\left(\mathrm{unrel}\right)}$
**Type II questions**	
-*Number of men in Horda County (D)*	*Progressive/successive presentation*: a hidden (AVH) commentator, while relevant images/sketches are displayed
-*Paid tribute to the Norwegian King (D)*-*Characteristics of the first phase of external territorial assembly (CU)*-*Consequences for the national assembly process when the Southern Isles and Man were rejected (CU)*	(VI_(rel)1_) as well as significant factual information successively presented visually-verbally as text signs (VI_(rel)2_). The pictorial information appears progressively and simultaneously or shortly after given commentary by the commentator (shifted image/speech).
	
	${\mathrm{CLT}}_{\mathrm{Type}\;\mathrm{II}}=\mathrm{AVH}\ldots {\mathrm{VI}}_{\left(\mathrm{rel}\right)1}\ldots {\mathrm{VI}}_{\left(\mathrm{rel}\right)2}$
Type III questions	
-*Gulating’s first time position (D)*-*Other National Kingdoms Combat King (D)*-*Party at the royal estate in Bergen (D)*.-*Kong Ola Haraldsson’s significance for the second phase of the national assembly (CU)*	*Multipresentation*: a hidden commentator tells thematic information (AVH) while showing relevant sketches, drawings, objects, or dramatizing representations (VI_(rel)3_) with background sound (AVB). Type III differs from Type II in that Type III does not have a similar progressive presentation and has no sign, and pictorial information has a somewhat lower degree of relevance than Type II.
	${\mathrm{CLT}}_{\mathrm{Type}\;\mathrm{III}}=\mathrm{AVH}+\mathrm{AVB}+{\mathrm{VI}}_{\left(\mathrm{rel}\right)3}$

The questions are marked whether they represent details (D) or connections/understanding (CU) in relation to the form of the teaching material.

**Table 2:** Overview of STM capacity categories and various STM-types (substructures) with associated test sets and concepts.

Capacity category	Description	Capacity STM-types (substructures)	Description
Progressive capacity	PC	PCvi + PCvv	Capacity measures on visual and verbal simultaneously and successively increasing amount of information
Multi capacity	MC	MCviP + MCviF + MCvvR	Capacity measurement of visual and verbal concurrent information
Sensory capacity	SC	SCvi + SCvv	Capacity measure of visual and verbal glimpses (1–2 s) simultaneously and increasing amount of information
Visual channel capacity	Visual	PCvi + MCviF + SCvi	Several types of visual capacity in a single target
Verbal channel capacity	Verbal	PCvv + MCvvR + SCvv	Several types of verbal capacity in a single target
Raven (extract RAPM)	Raven	Twelve matrices	Capacity for increasing non-verbal complexity

Capacity STM-types (substructures): PCvi, progressive visual-iconic; PCvv, progressive visual-verbal; MCviP, visual-iconic capacity picture; MCviF, visual-verbal multi capacity figure; MCvvR, visual-verbal recognition capacity; SCvi, visual sensory capacity; SCvv, verbal sensory capacity; Raven, non-verbal intelligence (extract). RAPM, Raven’s Advanced Progressive Matrices (12 selected).

**Table 3:** Overview of the sub-tests (capacity STM-types/substructures) included in the STM capacity measurement instrument.

Test	Capacity STM-type (substructures)	Designation	Description of capacity measurement
1	Progressive visual-iconic	PCvi	Successive increase in the number of simultaneous visual stimuli (figurer)
2	Progressiv visuell-verbal	PCvv	Successive increase in the number of simultaneous verbal stimuli (two-phoneme words)
3	Visual-iconic capacity picture	MCviP	A detailed realistic image (photograph) followed by statements (true/false) about details in the image to be remembered
4	Visual-iconic multi capacity figure	MCviF	A collection of simple figures is displayed at the same time and must be remembered
5	Visual-verbal capacity	MCvvR	A collection of two-phoneme words appears simultaneously and must be remembered
6	Visual sensoric capacity	SCvi	Successive increase in the number of simultaneous visual stimuli (figures) appears briefly (1–2 s)
7	Verbal sensoric capacity	SCvv	Successive increase in the number of simultaneous verbal stimuli (two-phoneme words) appears briefly (1–2 s)
8	Non-verbal intelligence (extract)	Raven (RAPM)	Raven matrices with increasing degree of difficulty is shown and the next logical pattern will be identified among several possibilities during the given viewing time per matrix

## Theoretical and Empirical Basis

With a theoretical basis in CLT, a number of scientific findings have been made that show multimedia has certain benefits for LOs, depending on how information is presented. For example, each of 10 principles of Mayer (2008) was based on a number of empirical studies (from 1991 to 2003), and the theoretical basis was anchored in the working memory’s limited capacity and integrated dual-code hypothesis. Integrated two-coding involves the simultaneous processing of information between visual and verbal channels (based on the classical dual-coding theory, DCT) in STM (Baddeley, 1986, 1999; Clark and Paivio, 1991). Recent models and studies on DCT have also shown that the interaction between visual and verbal channels is actually valid in all types of information processing, even where the stimuli are primarily given in only one of the forms of representation. A non-stimulated channel, thus, supports a stimulated channel with verbal or visual/iconic associations during the processing process (Kanellopoulou et al., 2019; Liu et al., 2020; Zhao et al., 2020). This processing contributes to the different representation forms that complement each other toward more robust and comprehensive learning. Such more integrated and interactive models are, thus, referred to as integrative models of text and picture comprehension (ITPC models; Schnotz, 2014; Schnotz and Wagner, 2018).

An integrated presentation of an educational material through multiple simultaneous representations will provide a better LO compared with a *successive* presentation of different forms of representation given gradually. For studies on LOs from multimedia compared with text, there are four specific principles that provide guidelines for the composition of different representation forms. These are as follows (Mayer, 2008, p. 376):

1. The Multimedia Principle: People learn better from words and pictures than from words alone [based, in part, on studies by Mayer and Anderson (1991, 1992) and Moreno and Mayer (1999, 2002)], with an effect size of Cohen’s *d* = 1.67.
2. The Contiguity Principle: People learn better from words and pictures of mutual relevance that are presented simultaneously rather than successively [based, in part, on studies by Mayer and Anderson (1991, 1992), Mayer and Sims (1994) and Mayer et al. (1999)], with an effect size of Cohen’s *d* = 1.3.
3. The Coherence Principle: People learn better if the presentation is free from insignificant extraneous information, such as background/noise [based, in part, on studies by Moreno and Mayer, 2000a,b) and Mayer et al., 2001], with an effect size of Cohen’s *d* = 0.96.
4. The Redundancy Principle: People learn better from animation and verbal comments than from animation, comments, and on-screen text [with part of the comments; based, in part, on studies by Mayer et al. (2001) and Moreno and Mayer (2002)], with an effect size of Cohen’s *d* = 0.69.

The studies based on these principles were made with relatively few respondents (approximately *n* = 30), with Mayer using Cohen’s *d* (Cohen, 1998) as a measurement without correction for the difference in SDs between the groups. This might have contributed to the high effect results. The test material mainly consisted of technical subjects with problem-solving as knowledge goals. The material was also limited in scope, with a composition specifically designed as a PC program with short sequences of different presentation forms (30–60 s). This may have contributed to an artificial learning situation for the respondents. Prior or subsequent thematic information, which is normally included in a training program in order to present a whole, has not influenced the STM capacity and LO in these test sequences. The individual differences in STM capacity, knowledge about the test subject, and other cognitive characteristics of the respondents were not included in these studies. Therefore, we do not know if the STM capacity contributed to the differences in LO between groups, although this formed the basis for the theoretical models in the experiments.

Central to Mayer’s *Context Principle* and *Redundancy Principle* is the importance of the *degree of relevance* between the information given, simultaneously or successively, between the different channels set up in the STM capacity. Previous studies have shown that a low relevance degree (cue summation) between information given simultaneously in the visual and verbal channels results in lower LOs. This is because of both a low degree of relevance and the limited capacity of STM to process simultaneous information (Hartman, 1961; Travers, 1964; Severin, 1967; Thesen, 1969; Nugent, 1982; Sadoski and Paivio, 2001). Recent multimedia-oriented design studies have shown that this is not necessarily the case (Salisbury, 1990; Sweller et al., 1998; Kalyuga et al., 1999; Johnson and Mayer, 2009). The split attention effect (split-attention effect; Chandler and Sweller, 1992; Mousavi et al., 1995; Tarmizi and Sweller, 1998; Ayres and Sweller, 2005; Owens and Sweller, 2008; Florax and Ploetzner, 2010, see also Sæverot and Torgersen, 2016) can partly compensate for limited STM capacity, so that an integrated composition of text, audio, and video can provide better LOs than presentations with separate or *progressive* information. These recent studies have shown that the capacity is not necessarily overloaded by *relevant* presentation through both visual and verbal channels simultaneously. In fact, an integrated presentation may contribute to information being processed more thoroughly by the information loop between the channels and, thus, provide stronger memory traces and better LOs. This requires, as both the classic studies and Mayer’s correlation and redundancy principle specify, that the information presented is mutually relevant.

A study by Florax and Ploetzner (2010) compared the LOs of five different compositions of text and pictures from a teaching material in neurology (the Synapse process). The study was based on five groups of students (each consisting of 33 students). The composition consisted of five different combinations between structured/unstructured text and *relevant* labeling/non-labeling of a picture. The five compositions were as follows: (1) continuous text + clean image; (2) textured text + clean image; (3) continuous text + image with arrows without text; (4) textured text + image with arrows and number reference to text; and (5) integrated = all in conjunction with (i) the image, text, arrows, and number (sign). The LO was measured with a knowledge test requiring both detailed knowledge and understanding (contexts). The material was presented through a digital screen to each of the groups but with different combinations. The study showed that integrated presentations provided the best LO, which supports the theory of a shared attention effect and that compound compositions do not necessarily overload the STM capacity (and thus inhibit learning). However, the relevance degree between the visual and verbal information was clearly high and particularly easy to identify and classify as high. Furthermore, in this study, only a static image was used, so it is uncertain what the learning results would be if the material had been presented with moving images (multimedia) and integrated text/labeling, which is common in educational software.

The study shows no difference in LO between the five compositions and plain text, only differences in LO between the five experimental measures. Studies by Furnham et al. (1990) show, for example, that pure text provided a significantly better LO than both speech and audiovisual presentations (video/multimedia). This applied to both simple and complex learning materials, and both free recall and cued recall (Furnham et al., 1990, p. 207).

The ratio of LOs was as follows, where > indicates a greater LO than: P (print, *n* = 19) > AV (film, *n* = 16) > A (auditory/talking head, *n* = 25), where free recall, *F* = 13.72 ∗∗∗; cued recall, *F* = 7.96 ∗∗; easy, *F* = 7.86 ∗∗; hard, *F* = 5.66 ∗∗, ∗∗∗ *p* < 0.001; ∗∗ *p* < 0.01, *n* = 60 (Furnham et al., 1990, p. 207).

If the different types of information coming through the various channels of content are irrelevant to each other, the LO is reduced (Brashears et al., 2005). Simultaneous irrelevant information through multiple channels can contribute to “Stroop effect” (cf. Stroop, 1935), where the different types of information mutually interfere with each other (Schmidt and Besner, 2008). Such processing puts far more strain on the cognitive process and challenges the STM capacity more than when the information is mutually relevant. In addition, interfering information can also weaken the clarity of the total information, which can impair the precision or accuracy of LO in relation to the original given information.

It is reasonable to assume that comprehensive (realistic) training multimedia provides a higher cognitive load compared with a single static image with text or just plain text. It is also difficult to assess the relevance degree between the information provided by such materials through various channels. However, a study by Koroghlanian and Sullivan (2000) shows that different verbal load density, through various compositions of auditory-verbal (speech) and visual-verbal (text) representations, does not result in significant differences in LO. On the other hand, when images are related to the verbal information, the composition and verbal form provide slight significance in LO. It is only when the images are connected to the verbal information that the composition and verbal form have any significant impact on the LO (cf. Mayer’s multimedia principle). Labeling may, for example, be a means to both capture (a part of) the attention toward significant areas (sections) of an image or a movie and provide additional details or explanations. Several studies have also shown, especially in news reporting, that labeling contributes to better LOs compared with communication that does not use labeling (Höier and Findahl, 1984; Erhel and Jamet, 2006; Mautone and Mayer, 2007).

These studies have shown that integrated double-coding processes in the STM do not necessarily impair the LO, but they did not demonstrate how the relationship and LO would have been if only text had been used with the same learning materials. Some of the studies, particularly Mayer’s basis for multimedia principles, have also used specially adapted learning materials with high or identical content relevance concerning various representation forms, as well as short and direct information sequences without these being part of an overall holistic presentation. However, a holistic presentation is normal and necessary in realistic training multimedia and multimedia presentations of learning materials for building a deeper understanding, as well as for emphasizing the meaning of the details (Woo Lee et al., 2008). These studies did not investigate how such difference in CLS affects LO, i.e., the extent to which there is a correlation between CLS and LO. Furthermore, none of these studies showed the importance of STM capacity for LO with various instruments in multimedia or pictures.

### The Main Objective of the Study

The main objective of this study is to investigate the importance of the three instruments in multimedia for LOs in relation to individual differences in STM capacity, where common simultaneous disturbances can also occur (e.g., sudden noise from other participants, for instance, coughing, movements, paper crackling, and several people in the same room).

#### Research Objectives

In order to investigate this main objective, we developed five specific research objectives in this study, which aim to examine the following:

1. The difference in LO between multimedia and text by three types of instruments in multimedia (comments, successive presentation, and multi-presentation).
2. Individual differences in the STM capacity and LO from multimedia with the three instruments.
3. The relationship between the capacity of various STM types (substructures) and the LO from multimedia with the three instruments.
4. Individual differences in channel capacity and LO from multimedia with the three instruments.
5. The relationship between CLS and LO based on the results of objectives 1–4.

The relationship between CLS and LO, that is research objective 5, is expressed schematically *via* the CLS-LO formula, which is introduced in this study.

## Concepts: Cognitive Load Structure and Instruments in Multimedia

Three instruments in realistic and digitalized training multimedia were selected as a basis for the examination of differences in LO between these and individual differences in the STM capacity. The narration of multimedia was transcribed into a text, which accounted for the text material in the study. The word *instrument (or tool)* is meant to cover the composition or composition forms of representation in multimedia, such as comments or voice, image, or labeling. As the voice and text contents of multimedia were identical, the image composition posed the difference in the two presentation forms. The image-related composition may also be combined in a particular relationship with the speech in the multimedia (comments). The composition of the various representation types in this study is defined as CLS. Based on CLS and the limited capacity of STM, there is not necessarily a correlation between CLS and LO. This relationship was, therefore, of theoretical and methodological interest in this study.

### The Instrument Effect

The sequences in the multimedia or text presentation, which provided information for knowledge questions, were referred to as *information sequences*. The difference between the LO for an information sequence with corresponding instruments in multimedia and the LO concerning the same information sequence given as text was defined as an *instrument effect*. The term “instrument effect” is not used directly in this study, but it is used as a theoretical factor in the design models. In the analysis, the term “difference in learning outcomes” is used to cover this. When interpreting the empirical results, the term capacity to *exploit* the instrument is also used. This refers to the capacity to utilize the way the instrument presents the learning material and how the representation forms (voice, text, and images) are composed together to convey the learning material. A better performance on knowledge tests for multimedia, but not for text, concerning students with higher STM capacity compared with students with lower capacity, could be interpreted to mean that the students with higher STM capacity utilize instruments from multimedia better than text as a presentation form. If LOs are similar between multimedia and text on the same information sequence, for example, for students with high STM capacity, it may be interpreted to mean that high STM does not contribute to the utilization of the instrument, or that high capacity is not sufficient to exploit the current composition of the representation forms so that LO improves.

The questions with information sequences that were included in various instrument types were also scattered in relation to the position in the multimedia and text material. Moreover, question progress and the position of the information sequences were similar for both multimedia and text, so a possible *primacy* or *recency* position could not have any effect *between* the two forms of presentation (Postman and Phillips, 1965; Tam and Ward, 2000).

The multimedia composition was not specifically designed for this study but was an actual multimedia educational program in Norwegian history. Based on the theoretical basis of this study, certain sequences were defined by specific measures based on the original composition of multimedia. Thus, the instruments are not identically conducted within the same tool types in the different information sequences but do have approximately the same composition and sequence duration.

### CLS-LO Formula

In the following, we introduce the CLS-LO formula. The purpose is to describe complex relationships between CLS and LO in a concise way. It can facilitate the study with theoretical analyses and models, and be of pedagogical contribution to the dissemination of complex findings in teaching and discussions of CLT-based studies. In the CLS-LO formula, the mathematical ratios >, =, and < are used, like the character usage of Furnham et al. (1990), to indicate the relationship between LO and CLS. The direction arrow ➔ is used as a sign of “leading to,” here either theoretically or empirically justified, where the same instruments are related to each other in the relationship between CLS and LO. This form is used to briefly represent the relationship (but is also explained verbally in the text).

### Instrument Type I: Comment

This instrument consisted of a commentator who was filmed while giving a spoken academic presentation. The commentator was in the field. This field setting was academically relevant (in the relevant geographic place), but the comments were not directly related to the area. The instrument involves providing information through two channels simultaneously: the auditory-verbal (the comments) and visual-iconic (image of the commentator and of the background). The instrument is called a (simultaneous) sum of the three forms of representation but where both the image of the commentator and the background are considered irrelevant. As the image of the commentator is synchronous with voice and sound, these two representation forms are linked together (AVS). Expressed by the CLS-LO formula, the relationship is as follows:

$${\mathrm{CLS}}_{\mathrm{Type}\;I}=\mathrm{AVS}+{\mathrm{VI}}_{\left(\mathrm{unrel}\right)}$$

Where

- CLS_Type I_, CLS for type 1 instrument (commentator in the field);
- AVS, auditory-verbal information (relevant synchronous thematic speech given by a visible commentator); and
- VI_(unrel)_, visual-iconic irrelevant information (unrel), image of the background where the commentator is placed visually in the field, but neither the field nor the commentator itself is considered relevant in relation to the content of the speech (given by AVS).

For instrument Type I, the formula thus expresses that the respondents (subjects/learners) are *exposed* to CLS (CLS_Type I_), which consists of a composition with *synchronous* instruments: commentator in field that delivers speech, auditory-verbal relevant information (AVS), and a visual-iconic irrelevant background information [VI_(unrel)_].

### Instrument Type II: Progressive/Successive Presentation

This instrument consists of a hidden commentator (AVH) who made spoken comments directly related to the visual information provided on the screen. The visual information consisted of relevant pictures or sketches [VI_(rel)1_], as well as text labeling with fact-based keywords [VI_(rel)2_], which were also given verbally by the commentator right before the display of text labels. The instrument involved information given through three representation forms that were made successively (progressive), auditory-verbally (comments), visually-iconically (pictures and sketches), and visually-verbally (text labels). All the information is mutually relevant. The information was given *successively*, which means a skewed picture/voice and labeling signs showed up in stages immediately after the spoken information was given. The text signs had an emphasizing or repetitive function concerning the information that was given orally, that is, a delayed double coding. The term “progressive” or “successive” suggests that the information was provided in stages, a little at a time, or at different times, between the various forms of presentation, and this the stimulation of one channel at a time but with a phase of overlapping. In some phases, there was also simultaneous information provided through the three channels. Nevertheless, the main feature of the instrument was still progressive presentation, with *one* focused channel at a time. In most sequences, either voice or image comes first. The instrument is characterized by a variety of representational forms, but where they all have a high relevance and cognitive load:

$${\mathrm{CLS}}_{\mathrm{Type}\;\mathrm{II}}=\mathrm{AVH}\ldots {\mathrm{VI}}_{\left(\mathrm{rel}\right)1}\ldots {\mathrm{VI}}_{\left(\mathrm{rel}\right)2}$$

Where

- AVH, auditory-verbal information (relevant thematic speech given by the invisible (hidden) commentator);
- VI_(rel)1_, relevant (visual-iconic) photographs or sketches; and
- VI_(rel)2_, occasionally (visual-verbal/iconic) text-labels with fact-based keywords.

For instrument Type II, the formula, thus, expresses that the respondents (subjects/learners) are *exposed* to a CLS (CLS_Type II_), which consists of a composition with *successive* instruments: relevant thematic speech given by the invisible (hidden) commentator (AVH), relevant photographs or sketches [VI_(rel)1_], and, occasionally, text labels with fact-based keywords [VI_(rel)2_].

### Instrument Type III: Hidden Commentator With Relevant Background

Unlike Type I, this instrument consists of a hidden commentator (AVH), who provides factual information supported by relevant images and short footage/dramatizations [VI_(rel)3_] but without text signs. The sequences here were structured with a fast stage and image shifts along with the comments. The shifts took place within 2–5 s. In addition, there was background music and/or real sound (AVB). This composition was defined as a *multi-presentation*, which means that the information through the various forms of presentation was given simultaneously. The instrument consisted of three simultaneous presentations of high relevance between them, and, overall, the instrument has the highest CLS of the three types:

$${\mathrm{CLS}}_{\mathrm{Type}\;\mathrm{III}}=\mathrm{AVH}+\mathrm{AVB}+{\mathrm{VI}}_{\left(\mathrm{rel}\right)3}$$

Where

- AVH, auditory-verbal information (relevant academic speech given by the invisible (hidden) commentator);
- AVB, auditory-background music and/or real sound; and
- VI_(rel)3_, relevant pictures and short movie clips/dramatizations but without text signs.

For instrument Type III, the formula, thus, expresses that the respondents (subjects/learners) are *exposed* to a CLS (Type III), which consists of a composition with *many (multi) simultaneous* instruments: relevant thematic speech given by the invisible (hidden) commentator (AVH), background music and/or real sound (AVB), and relevant pictures and short movie clips/dramatizations but without text signs [VI_(rel)3_].

## Theoretical Assumptions Expressed with the Cognitive Load Structure-Learning Outcome Formula

In this section, we derive theoretical assumptions on the relationship between CLS in the three types of instruments we used in this study and on the expected LOs. We express these conditions with the CLS-LO formula (cf. research objective 5).

Based on Mayer’s four multimedia principles, one would expect here that LO would be best for type III, although this instrument has the highest CLS. The theoretical reason for this is that a shared attention effect helps to compensate for the load, and an integrated double-coding process will take place in the working memory because several forms of representation will convey the same information simultaneously or successively. Theoretically, it will create stronger memory traces of the information presented, and the respondent remembers this better.

In principle, type I and type II represent a *one* code-process, and therefore no differences were expected. Nevertheless, if one adds the *Redundancy Principle* (Mayer et al., 2001), Type I (which is based on spoken comments) will have an advantage in terms of LO compared with Type II, which in turn uses more representation forms. Type II is therefore considered to represent a slightly higher CLS than Type I. Based on these empirical and theoretical assumptions, the following relationship between CLS and LO is expected:

$${\mathrm{CLS}}_{\mathrm{Type}\;\mathrm{III}}>{\mathrm{CLS}}_{\mathrm{TypeII}}>{\mathrm{CLS}}_{\mathrm{Type}\;I}\to {\mathrm{LO}}_{\mathrm{Type}\;\mathrm{III}}>{\mathrm{LO}}_{\mathrm{Type}\;I}>{\mathrm{LO}}_{\mathrm{Type}\;\mathrm{II}}$$

The CLS-LO formula states that the CLS decreases from type III to type I, and the expected LO will be greatest with type III, somewhat less with type I, and least for type II.

However, because this study uses realistic learning materials as a basis, a somewhat different LO was expected compared with the referenced studies that underlie Mayer et al. (2001) multimedia principles. Theoretically, based on the CLT and an assessment of the information density of the three instruments, it is expected that the difference in the CLS between the instrument types in the multimedia used in the experiment would be as follows:

$${\mathrm{CLS}}_{\mathrm{Type}\;\mathrm{III}}>{\mathrm{CLS}}_{\mathrm{Type}\;I}>{\mathrm{CLS}}_{\mathrm{Type}\;\mathrm{II}}$$

The CLS-LO formula states that the theoretically derived CLS is highest for Type III, and that Type I entails a higher CLS than Type II.

In other words, instrument type III represents the multi-presentation, which consists of a higher simultaneous density of information than both Types I and II. Type II is considered to have higher information density than Type I, but because the information is presented successively, it is considered to correspond better with the processing capacity of the STM and thus does not challenge the STM capacity as much as Type I or Type III. Mayer (2009) also reports through his five studies that the image of a commentator in a video can provide higher cognitive load and lower LOs compared with a video without an image of the commentator (Cohen’s *d* = 0.22), “Image Principle” (Mayer, 2009, p. 260). It also supports the theoretical assumptions here in the relationship between Type I and the other two instruments. However, the effect size is weak, and the studies were conducted with a game-oriented learning material. Therefore, this principle will not be emphasized in the analysis and discussion of this study.

Consequently, in this study, it is expected that LOs from a presentation with Types I and II will be higher than from Type III. Since Type II stimulates STM successively, the information is processed in stages, thus enabling all the information to be processed thoroughly, and it is expected that Type II would provide a better LO than Type I. Based on this, the following relationship between the CLS and LO of the three instrument types are as follows:

$${\mathrm{CLS}}_{\mathrm{Type}\;\mathrm{III}}>{\mathrm{CLS}}_{\mathrm{Type}\;I}>{\mathrm{CLS}}_{\mathrm{Type}\;\mathrm{II}}\to {\mathrm{LO}}_{\mathrm{Type}\;\mathrm{II}}>{\mathrm{LO}}_{\mathrm{Type}\;I}>{\mathrm{LO}}_{\mathrm{Type}\;\mathrm{III}}$$

The CLS-LO formula states that the CLS is highest for Type III, which may contribute to this instrument providing the lowest or worst LO. Type II is theoretically considered to have a medium CLS but will give the highest or best LO. Type I will have a medium CLS and, theoretically speaking, contribute to a medium LO in this experiment.

## Materials and Methods

### Data Collection

Parts of the dataset used in this study have previously been described in a doctoral thesis at the Norwegian University of Science and Technology (NTNU; Torgersen, 2012) and in an article (Torgersen and Sæverot, 2016). These studies examined general LOs between multimedia and analogue text in relation to capacity types in STM, such as the meaning of verbal and visual channels. A partial study in Torgersen (2012) also examined the importance of various concrete tools in multimedia. This study continues these analyses in a broader learning context. A later study based on the data in this study will examine a possible overall position effect in light of STM capacity (serial position effect) for both multimedia and text (Torgersen and Boe, in press). In this later study, the forms of expression with the CLS-LO formulas will also be supplemented and further developed.

### Samples and Procedures

The total sample (*N* = 389) consisted of students at the undergraduate level, including military officers (*n* = 94, Norwegian Military Academy and the Norwegian Command and Staff College), student teachers (*n* = 194), and a mixed group of engineer and psychology students from the NTNU (*n* = 101). Eleven respondents are missing in the collected data set. The sample used contained 193 women and 185 men (*n* = 378). In this study, the academic field was not used as a variable. These were selected based on which classes had the opportunity to contribute to the experiment, chosen by the current head teacher at each school who had an overview of timetables at the schools. Some student groups at NTNU also became participants *via* notices about the opportunity to participate at certain times.

First, the STM test was conducted and everyone participated in the same test. The various classes conducted these in their own classroom/auditorium. Then, the respondents were divided into two groups, according to whether they were exposed to multimedia as a presentation (n_MM_ = 189) or to text (n_T_ = 189) as the control group. This division was made just before the experiment, based on a list of names where the first was placed in the MM group and the next in the text group. This was conducted in each class. The distribution was men and women, 99/88, respectively, for multimedia, and 94/97 for the text group. The overall response rate was 95.5%.

It was just the n_MM_ group that was tested on the three instruments in multimedia. Everyone in this group was exposed to the three tools. LOs (from the academic content material) were also tested for the control group (n_T_) with the same knowledge questions as the n_MM_ group. The answers were given in exactly the same positions in the teaching material for the n_T_ group as for the n_MM_ group. The experimental difference was that the learning material was produced with three different tools for the n_MM_ group, but for the control group (n_T_) the information was only presented as text (and had to be read). In this way, the possible effect of the instruments could be identified and controlled against text as a learning source, on the same material and with the same degree of difficulty. There were no significant differences in total STM capacity between n_MM_ and n_T_. However, there was a small but non-random difference in the Raven scores between the two groups. A one-way ANOVA showed that the difference between the groups was significant (*p* < 0.01, *F* = 7.023, and *p* = 0.008), but that the correlation was weak (*η*^2^ = 0.02), which gives an explained variance of 2%. That is, only 2% of the Raven scores can be explained based on which group (n_MM_ or n_T_) the test subjects belonged to. The level of Raven scores can, therefore, be considered to be approximately equal in both groups.

Even though the data collection and report were completely anonymous, an application for ethical consideration was sent to both the Norwegian Military Academy and Norwegian Command and Staff College to gain approval for the study. The study was approved by both institutions, and written informed consent was obtained from the participants of the study.

The survey was carried out in the regular classroom or lecture hall of the respondents and conducted in connection with a regular lesson. First, a brief (5 min) introduction was given, and forms for anonymity and informed consent were addressed. Then the STM test was conducted in plenary with the use of PowerPoint (about 20 min). The respondents checked their answers on the distributed form. Finally, the educational multimedia was seen or the text was read, and a knowledge test followed (about 20 min in total). The entire survey was completed in about 60 min.

### Measures

In this study, two main variables were measured. One was LO from the multimedia or text, and the other was the level of STM (category capacity and channel capacity; see Tables 2 and 3). The LO was measured with knowledge questions relevant to the information in the multimedia where the various representation forms were incorporated (Table 1). The learning content was about Norwegian history, more specifically the Unification Conflict (800–1,270 AD). The design was such that all the respondents first received the STM test and then received the learning test either *via* multimedia or analogue text. Everything took place in natural learning environments, such as classrooms and auditoriums (*n* = 25–60). Also, all the participants received a pre-knowledge test on the learning theme of the experiment a month before the experiment itself, which indicated that they had no advance knowledge of the topic given in the learning test.

### Measurement of Learning Outcomes

The LOs were measured in two samples. One group was exposed to a multimedia presentation, and another group received a text as a presentation form. The multimedia consisted of a selected sequence of 9 min and 15 s from an educational presentation that dealt with an era in Norwegian history, the Unification Conflict (800–1,270 AD; University of Bergen, 1990). The multimedia sequence was chosen in accordance with certain criteria, among others the three relevant instruments that were to be included in the study. The teaching material had to be relatively unknown to the test group and did not demand any prior knowledge of the subject (criteria of unfamiliarity). When testing and comparing LOs from various presentation forms, it is necessary to be certain that the knowledge is a result of the presentation and not prior knowledge of the subject (there are requirements for no prior knowledge of the testing theme in experimental comparative multimedia learning research, Torgersen and Vavik, 2005). Measured LOs must then be attributed to the presentation being made in the experiment.

The text material was identical to the narrative of multimedia, with a total of 1,113 words. The allotted time for text reading was 8 min and 25 s, which gave the same exposure time for both multimedia and text, based on a normal reading speed of about 140 words per minute. The LOs from both presentation forms were measured with a knowledge test consisting of 13 questions, where the answers were divided equally between the multimedia and the text. The positions of the information (answers) in the presentation were also identical between the multimedia presentation and the text.

The questions also measured the form of the teaching material or subject matter, and the difference between detail and context (understandings) was emphasized (Lund, 1991; Torgersen, 1999). Details meant knowledge about certain dates and names, and this was measured with nine questions. The knowledge that required context and further explanations was measured with four questions. The knowledge test offered five response options for each of the questions. All the response options were relevant to the subject, but only one of the five response alternatives was correct (multiple choice). The responses were only oriented toward the spoken information and were either just given verbally (also reproduced in the text) or both verbally and by labeling in the multimedia.

The LOs concerning the three instruments in the multimedia were measured by the questions in the knowledge test (Table 1). Since the multimedia was a regular training multimedia, it was not possible to define the same number of issues with the three instruments. All still contained at least one question that would measure connections/understanding and a minimum of three detailed questions. The questions that were to measure LO by related measures were considered equally difficult with a relatively equal presentation scale (explanation/description). The difference was, therefore, the use of instruments by the information sequences.

### Measure of Short-Term Memory Capacity and Channel Capacity

Previous studies on STM (cf. Doolittle and Altstaedter, 2009; Lusk et al., 2009) have used traditional test batteries, such as the Wechsler Memory Scale – Third Edition (WMS-III, Wechsler, 1997) and Operation Span Task (OSPAN-Task test, Turner and Engle, 1989).

However, these tests were developed before the age of multimedia; and it is unlikely they will grasp the multimodal forms of representations. They are, therefore, unable to capture specific STM-oriented processes when learning from modern multimedia and the diversity of multimodal forms of presentation. Therefore, Torgersen and Barlaug (2004) STM test was used to measure the STM capacity with sub-processes (capacity types or substructures) in STM (Tables 2 and 3). In this test, channel capacity is measured in all the visual and verbal STM tests of this measuring instrument. The period of residence, or processing time, as a definition of STM, is set between 2and 30 s, with SR set to interval 1–2 s, which is the basis for the construction of the STM test (cf. Howard, 1983; Conway et al., 2005; Cowan, 2005; Dehn, 2008). Other STM tests, such as the Wilde-Intelligence Test (Jäger and Althoff, 1983), are not nuanced enough and could therefore not be used to measue the different capacity types investigated in this study.

To measure capacity depending on how information is presented, specific tests were pooled. Three types of processing (capacity categories) were distinguished within the STM (Tables 2 and 3).

One was described as progressive capacity (PC), which measures the capacity to process a little information at a time (progressive presentation), where the scope is gradually increased (Tables 2 and 3). The second term was multi capacity (MC), which measures the capacity to process a considerable amount of information that is given simultaneously. In addition, the capacity to recall short information glimpses (1–2 s) was defined as a separate category and matches the classic model’s use of the term “Sensory Register/Memory” (SR). This was accordingly termed sensory capacity (SC).

Visual and verbal channel capacity is the capacity to process visual or verbal information, in which progressive, multi-oriented, and sensory forms of presentation are included. Each of these was also measured with specific subtests (capacity types or substructures, see Tables 2 and 3).

To investigate the importance of individual differences in STM capacity for LO, the targets for STM were divided into three capacity levels: *low*, *medium*, and *high*. The classification was based on quartile divisions or made on the basis of frequency distribution regarding the correct number on the STM tests. Both visual and verbal channel capacities were also divided into three capacity levels (low, medium, and high) for quartile divisions. The first category (low) consisted of approximately the first quartile, the other category (medium) approximately the second and third quartiles, and the third category (high) approximately the fourth quartile (see Torgersen, 2012).

All in all, the test battery contained eight test components with a total of 58 test stages, divided into the three capacity categories (progressive capacity, multi-capacity, and sensory capacity) including 12 Raven matrices with increasing difficulty for measuring non-verbal capacity and pattern recognition. The STM test was presented as a PowerPoint presentation from a large screen. The test was presented automatically with programmed time intervals and divided time breaks between the tests, from start to finish. The total duration of the test, including programmed information, was 1,076 s (17 min 56 s). A vision test (everyone had to see the large screen clearly) and the overall test information were also included. The respondents ticked off their answers to analogous answers in the individually distributed material.

### Statistical Analysis and Relative Values

In this study ANOVA, MANOVA, and regression (stepwise) were consistently used to examine differences in outcomes after instrument types and STM capacity. The different types of STM capacity were divided into three levels that approximate the first quartile (low), the second and third quartiles (medium), and the fourth quartile (high; Torgersen, 2012).

Torgersen (2012) showed that there was a *low* media-related difference in total LO between multimedia and text, where text was the best (*F* = 3.69, *p* < 0.05, Cohen’s *d* = 0.2). If learning from the actual instruments in the multimedia is compared with the corresponding questions for learning with text, based on average scores, the test scores were higher because the LO was, in general, better with the text in these studies. This was, therefore, corrected using relative values. The relative targets were calculated as follows: the number of correct answers in each of the groups with questions that measured the LO by means of Types I, II, and III was individually divided by the total number of correct answers (for the three instrument types), respectively, for multimedia and text. In this way, the various issues that monitor information relating to the instruments are compared across the types of multimedia and text. The difference between LOs, expressed in relative values, from multimedia and text, is used when the goal is to measure the effect. The average number of correct answers (*M*) was also stated under each instrument type.

## Results

### Research Objective 1: Differences in Learning Outcome Between Multimedia and Text Measured by Three Types of Instruments in Multimedia

Table 4 shows the difference in LO between multimedia (*n* = 189) and text (*n* = 189) with an ANOVA. MM*r* and T*r* indicate the relative values of the LO from multimedia and text measured with the three instruments, where a possible improved LO related to media is considered where the text is best. MM*M* and T*M* show the mean values for multimedia and text with the three instruments. The table shows that in the questions where the information was related to the sequences, in which type I was included in the multimedia, the difference in LO between text and multimedia was significant (*F* = 7.63, *p* < 0.01, *η*^2^ = 0.02) to the advantage of text. In relation to the text, the instrument in multimedia had little or no effect.

**Table 4:** Relative and mean values of learning outcomes from multimedia (MM) compared to text (T).

	Type I	Type II	Type III
**MM**			
MM*r*^1^	0.33	0.36	0.30
MM*M*^2^	2.30	2.51	2.14
**Text**			
T*r*^3^	0.38	0.30	0.32
T*M*^4^	2.82	2.23	2.38
*F*	7.63^∗∗^	16.25^∗∗∗^	0.69
*η*	0.14	0.20	0.04
*η*^2^	0.02	0.04	0.04

Learning outcomes (MMM/TM) are indicated on a scale where 0 is worst and 5.0, 4.0, and 4.0 are best for Type I, Type II, and Type III, respectively. The overall average learning outcomes is 6.95 (out of 13.0) for multimedia and 7.43 for text.

1 MM*r*, Multimedia relative value.

2 MM*M*, Multimedia mean value.

3 T*M*, Text mean value.

4 T*r*, Text relative value.

∗ *p* < 0.05;

∗∗ *p* < 0.01;

∗∗∗ *p* < 0.001.

In contrast, the pattern in type II is the opposite. The highest results for LO in this type were for multimedia (*F* = 16.25, *p* < 0.001, *η*^2^ = 0.04). This may be due to the *η*^2^ value of the Type II instrument (*η*^2^ = 0.04), which also shows that approximately 4% of the differences in LO between multimedia and text can be explained by whether the respondents had multimedia or text. For type III, there was no difference between multimedia and text. Furthermore, there was no significant difference in LO between instrument types within the same presentation (multimedia and text). However, LO was best for text Type 1. This may indicate that the questions or subject matter relating to this part of the learning material was easier than the others. If this is the case, it may indicate an additional effect of Type II, where the LO was lowest for text and best for multimedia. If these questions and the subject matter here were difficult, it may suggest that instrument type II in the multimedia contributed further to a better LO with multimedia.

### Research Objective 2: Individual Differences in the Short-Term Memory Capacity and Learning From Multimedia With Three Instruments

Table 5 shows that there was a rise in LO for the individuals with high STM capacity for both multimedia and text. There were significant differences in LO between multimedia and text for the individuals with medium STM capacity (*F* = 11.5, *p* < 0.001, *η*^2^ = 0.06) in the type I instrument in multimedia. For the individuals with low and medium capacity, STM learning proceeded best by text. On the other hand, for the individuals with high-capacity STM, there was no difference in outcome between text and multimedia. What was special about the type I instrument was that the difference in outcome between multimedia and text disappeared for the individuals with high-capacity STM. This may indicate that instruments in type I had the greatest impact on the individuals with high-capacity STM while learning from text. This is because the difference in LO between multimedia and text disappears for the individuals with high-capacity STM. This may further support the notion that the individuals with high-capacity STM better utilize the type I instrument for learning. The individuals with low and medium STM capacity learned most from text, which may indicate that an instrument such as type I can actually be a learning problem for individuals with low or medium STM capacity.

**Table 5:** Relative values for learning outcomes from multimedia (MM*r*) with three types of instruments and text (T*r*) for three STM capacity levels.

STM	Type I			Type II			Type III		
	Low	Medium	High	Low	Medium	High	Low	Medium	High
MM*r*^1^	0.27	0.33	0.41	0.31	0.37	0.39	0.28	0.30	0.35
*n*	50	97	45	50	97	45	50	97	45
T*r*^2^	0.31	0.42	0.39	0.25	0.32	0.32	0.29	0.33	0.34
*n*	55	89	48	55	89	48	55	89	48
*F*	0.80	11.50^∗∗∗^	0.30	4.09^∗^	6.04^∗^	6.61^∗^	0.24	1.46	0.28
*η*	0.13	0.24	0.06	0.20	0.18	0.26	0.05	0.09	0.06
*η*^2^	0.02	0.06	0.00	0.04	0.03	0.07	0.00	0.01	0.30

1 MM*r*, Multimedia relative value.

2 T*r*, Text relative value.

∗ *p* < 0.05;

∗∗ *p* < 0.01;

∗∗∗ *p* < 0.001.

For type II, multimedia received the highest score at all STM capacity levels. The biggest difference between multimedia and text was for individuals with high STM capacity, with the advantage of multimedia being (*F* = 6.61, *p* < 0.05, *η*^2^ = 0.07). This may indicate that STM capacity did not have any impact on LO, which means that everybody could benefit from this instrument. For type III, there were no significant differences in LO between multimedia and text. This may indicate that the instrument does not *inhibit* learning either.

### Research Objective 3: The Relationship Between the Capacity of the Short-Term Memory Types (Substructures) and Learning Outcome From Three Instruments in Multimedia

In order to examine more specifically the STM types that are related to LO from the three instruments in multimedia, linear regression models with a stepwise procedure were used. Table 6 shows that the progressive visual-verbal capacity (PCvv) was related to LO from multimedia. This was the case for instrument Type I (*β* = 0.21, *p* < 0.01, *R*^2^ = 0.04) and type III (*β* = 0.2, *p* < 0.01, *R*^2^ = 0.04). However, the strongest correlation was between Raven and LO through instrument type II (*β* = 0.27, *p* < 0.001, *R*^2^ = 0.07). Concerning text, there were other STM types that were related to LO besides those that applied for multimedia. This was measured with the same questions that focused on LO through the three instruments in multimedia (information sequence). This supports the model of a learning effect from multimedia, as the regression analysis shows. In general, there were weaker *β*-values for text than for multimedia. However, it is important to note that the explained variance in these analyses is relatively low, and although some predicting variables provide significant contributions, they are modest.

**Table 6:** Stepwise regression analysis for the capacity of various STM-types (substructures) and learning outcomes from three instruments in multimedia (relative values).

STM-types	MM			Text		
	Type I	Type II	Type III	Type I	Type II	Type III
	*β*-value	*β*-value	*β*-value	*β*-value	*β*-value	*β*-value
PCvi						
PCvv	0.21^∗∗^		0.20^∗^			
MCviP						
MCviF						0.14^∗^
MCvvR				0.15^∗^	0.19^∗∗^	
SCvi				0.18^∗^	0.17^∗^	0.20^∗∗^
SCvv						
Raven		0.27^∗∗∗^		0.11^∗^		0.19^∗^
*R*	0.21	0.27	0.20	0.30	0.27	0.33
*R*^2^	0.04	0.07	0.04	0.09	0.07	0.11
R^2^ adj.	0.04	0.07	0.03	0.06	0.06	0.10

As the instrument is not found in text, but questions and answers can still be found here, learning outcomes are given by the same information sequences with I, II, and III.

∗ *p* < 0.05;

∗∗ *p* < 0.01;

∗∗∗ *p* < 0.001.

In addition to Raven, it was only PCvv that related to LO from the instruments. PCvv was, therefore, examined in relation to low, medium, and high-capacity levels for the three instrument types. Achievements on the STM tests from progressive visual capacity (PCvi) were transformed to three capacity level categories: low (28.8%), medium (25.1%), and high (36.1%); and similarly for PCvv: low (23%), medium (41.2%), and high (35.9%).

Table 7 shows that for type I the average values for LO from the three capacity levels were consistently higher from text than from multimedia. However, the difference between the capacity levels were greater for multimedia (*F* = 7.11, *p* < 0.001, *η*^2^ = 0.07) than for text (*F* = 3.96, *p* < 0.05, *η*^2^ = 0.04). In particular, the individuals with low PCvv had very low scores on LO from type I (*M* = 1.71), compared with text (*M* = 2.37). For the individuals with medium and high PCvv, the difference in LO between the three capacity levels was less compared with text. This may indicate that the type I instrument, which consisted of an audio-verbal presentation, did not promote LO compared with a purely visual-verbal presentation. The individuals with low PCvv also had the lowest LO from this instrument (audio-verbal presentation).

**Table 7:** Mean values (non-relative values) for learning outcomes for verbal progressive capacity (PCvv) for the three instruments in multimedia (MM).

PCvv	MM				Text			
	Type I	Type II	Type III	*n*	Type I	Type II	Type III	*n*
Low	1.71	2.15	1.82	39	2.37	1.92	2.13	38
Medium	2.38	2.60	2.14	130	2.89	2.21	2.44	127
High	2.87	2.61	2.65	23	3.19	2.74	2.41	27
*F*	7.11^∗∗∗^	3.04^∗^	4.21^∗^		3.96^∗^	4.45^∗^	1.50	
*η*	0.27	0.18	0.21		0.20	0.21	0.13	
*η*^2^	0.07	0.03	0.04		0.04	0.05	0.02	

As the instrument is not found in text, but questions and answers can still be found here, learning outcomes are given by the same information sequences with I, II and III.

∗ *p* < 0.05;

∗∗ *p* < 0.01;

∗∗∗ *p* < 0.001.

There were significant differences in LO between capacity levels in both types II and III. Average values show that LO was higher for multimedia in which the type II instrument was used for those with low and medium PCvv compared with the corresponding text. This may indicate that the type I instrument, which consists of real comments (auditive-verbal presentation) did not promote LO compared to a pure visual-verbal presentation. The individuals with low PCvv also had the lowest LO from this instrument.

There were significant differences in LO between the capacity levels for types I and II. The average values show that LO was higher for multimedia where the Type III instrument was used for the individuals with low and middle PCvv compared with a similar situation for text.

An ANOVA of the PCvi was also conducted, even though it did not register on the regression analysis. Table 8 shows that there were significant differences in outcomes between the three capacity levels for types I (*F* = 3.28, *p* < 0.05, *η*^2^ = 0.03) and II (*F* = 4.65, *p* < 0.05, *η*^2^ = 0.05).

**Table 8:** Mean values (non-relative values) for learning outcomes for visual progressive capacity (PCvi) for the three instruments in multimedia (MM).

PCvi	MM				Text			
	Type I	Type II	Type III	*n*	Type I	Type II	Type III	*n*
Low	2.07	2.17	1.95	41	2.42	1.80	2.00	45
Medium	2.24	2.51	2.14	114	2.88	2.34	2.43	117
High	2.76	2.86	2.32	37	3.23	2.43	2.72	30
*F*	3.28^∗^	4.65^∗^	1.10		4.20^∗^	4.59^∗^	5.32^∗∗^	
*η*	0.18	0.22	0.11		0.21	0.22	0.24	
*η*^2^	0.03	0.05	0.01		0.04	0.05	0.06	

As the instrument is not found in text, but questions and answers can still be found here, learning outcomes are given by the same information sequences with I, II and III.

∗ *p* < 0.05;

∗∗ *p* < 0.01;

∗∗∗ *p* < 0.001.

### Research Objective 4: Individual Differences in Channel Capacity and Learning From Multimedia With Three Instruments

To investigate the importance of visual and verbal channel capacity for learning from multimedia, an ANOVA was conducted. Category text was also used here as a reference to compare with LO from multimedia in which the same instruments were included. Consequently, any difference in LO may be attributed to this instrument.

Table 9 shows that for the visual channel there were significant differences in LO between multimedia and text by means of type II for all the three-channel capacities (*p* < 0.05). The LO was also highest for multimedia compared with the corresponding text for both individuals with low, medium, and high visual channel capacity. This may indicate that instrument type II had a learning-enhancing effect compared to text, regardless of channel capacity. For instrument type I, the situation was just the opposite. Here, text was best in terms of LO for all the channel capacities but with a significant difference only for the individuals with moderate visual channel capacity (*F* = 8.18, *p* < 0.01, *η*^2^ = 0.03).

**Table 9:** Differences in learning outcomes (relative values) between multimedia (MM*r*) and text (T*r*) for the three instruments for visual channel capacity.

Visual	Type I			Type II			Type III		
	Low	Medium	High	Low	Medium	High	Low	Medium	High
*n*	87	239	70	87	239	70	87	239	70
MM*r*^1^	0.30	0.32	0.40	0.31	0.36	0.41	0.28	0.31	0.33
T*r*^2^	0.33	0.39	0.44	0.24	0.33	0.33	0.27	0.33	0.37
*F*	0.47	8.18^∗∗^	1.22	5.32^∗^	5.75^∗^	5.92^∗^	0.12	1.12	0.92
*η*	0.05	0.19	0.14	0.24	0.16	0.29	0.04	0.07	0.12
*η*^2^	0.01	0.03	0.02	0.06	0.03	0.08	0.00	0.00	0.01

*n*, indicates respondents within the channel levels.

1 MM*r*, Multimedia relative value.

2 T*r*, Text relative value.

∗ *p* < 0.05;

∗∗ *p* < 0.01;

∗∗∗ *p* < 0.001.

This may suggest that instrument Type I did not promote learning in relation to text. For the individuals with average channel capacity, the results also show that this instrument inhibited learning. For Type III, there was no significant difference in LO between multimedia and text within the three-channel capacities. Based on average values of the relative sizes, LO was the same for the individuals with low capacity, and text was best for the others. This may indicate that instrument Type III also had no learning-enhancing effect compared with text.

Concerning the verbal channel capacity, the patterns were essentially the same for visual channels, with some exceptions. Table 10 shows that the LO was generally better with learning from text, except with instrument Type II. Here, the LO was generally better from multimedia than from text, and it was especially the individuals with moderate verbal channel capacity that learned the most from multimedia compared with text (*F* = 17.53, *p* < 0.001, *η*^2^ = 0.06).

**Table 10:** Differences in learning outcomes (relative values) between multimedia (MM*r*) and text (T*r*) for the three instruments for verbal channel capacity.

Visual	Type I			Type II			Type III		
	Low	Medium	High	Low	Medium	High	Low	Medium	High
*n*	80	263	53	80	263	53	80	263	53
MM*r*^1^	0.25	0.34	0.41	0.31	0.37	0.38	0.26	0.31	0.38
T*r*^2^	0.32	0.39	0.43	0.26	0.30	0.37	0.29	0.33	0.32
*F*	3.48	4.53^∗^	0.15	2.34	17.53^∗∗∗^	0.02	0.55	1.35	2.06
*η*	0.21	0.13	0.06	0.17	0.25	0.02	0.09	0.07	0.20
*η*^2^	0.04	0.02	0.00	0.03	0.06	0.00	0.01	0.01	0.04

*n*, indicates respondents within the channel levels.

1 MM*r*, Multimedia relative value.

2 T*r*, Text relative value.

∗ *p* < 0.05;

∗∗ *p* < 0.01;

∗∗∗ *p* < 0.001.

To sum up, the results show that it was instrument Type II that promoted LOs best from multimedia compared with text and for both visual and verbal channel capacities in general. Individual differences in channel capacity had little effect on the ability to utilize the learning instruments.

### Research Objective 5: The Relationship Between Cognitive Load Structure and Learning Outcome: Cognitive Load Structure-Learning Outcome Formula

The aim of research objective 5 was to discuss and express the relationship between CLS and LO based on the results of research objectives 1–4. Based on previous empirical studies and the theoretical foundation that this study builds on, the main assumption is that the relationship with respect to CLS and LO is the following, expressed with the CLS-LO formula:

$${\mathrm{CLS}}_{\mathrm{Type}\;\mathrm{III}}>{\mathrm{CLS}}_{\mathrm{TypeII}}>{\mathrm{CLS}}_{\mathrm{Type}\;I}\to {\mathrm{LO}}_{\mathrm{Type}\;\mathrm{III}}>{\mathrm{LO}}_{\mathrm{Type}\;I}>{\mathrm{LO}}_{\mathrm{Type}\;\mathrm{II}}$$

Given that this study was based on a comprehensive teaching material, the theoretical ratio was adjusted to the following:

$${\mathrm{CLS}}_{\mathrm{Type}\;\mathrm{III}}>{\mathrm{CLS}}_{\mathrm{Type}\;I}>{\mathrm{CLS}}_{\mathrm{Type}\;\mathrm{II}}\to {\mathrm{LO}}_{\mathrm{Type}\;\mathrm{II}}>{\mathrm{LO}}_{\mathrm{Type}\;I}>{\mathrm{LO}}_{\mathrm{Type}\;\mathrm{III}}$$

The relationship of CLS is maintained based on the results of this study. However, the consequences for LO were not as expected. The LO for instrument Type II was *higher* than for both Types I and III, while there was no difference between Types I and III. Thus, on the basis of this study, the relationship is as follows:

$${\mathrm{CLS}}_{\mathrm{Type}\;\mathrm{III}}>{\mathrm{CLS}}_{\mathrm{Type}\;I}>{\mathrm{CLS}}_{\mathrm{Type}\;\mathrm{II}}\to {\mathrm{LO}}_{\mathrm{Type}\;\mathrm{II}}>{\mathrm{LO}}_{\mathrm{Type}\;I}={\mathrm{LO}}_{\mathrm{Type}\;\mathrm{III}}$$

The CLS-LO formula expresses the empirical result from this study: Type II was considered to have the lowest CLS. The highest LO was measured with this instrument. Types I and III gave almost equal LOs, even though the theoretically derived CLS was considered to be higher for Type III than for Type I. Of the three instruments, Type II was, thus, best in relation to LOs in general.

Mayer’s (2005) multimedia principle was based on studies where the educational material was quite simple, with only short information sequences as learning material, and these sequences were not part of the overall presentation. This may imply that integrated presentations provide the best LO as long as the learning material is easy. When the information is presented as a whole, it may increase the cognitive load, thereby reducing the LO because of STM capacity. Other studies on complex and comprehensive learning materials also support such relationships (Furnham et al., 1990).

## Discussion

The main objective of this study is to investigate the importance of the three instruments in multimedia for LOs in relation to individual differences in STM capacity. The main finding was that the *successive* presentation (Type II instrument) consisting of learning materials with several representation forms (voice, images, and text) provided better LO than just text for those with low, medium, and high STM capacity. This matched well with the expected results based on CLT and the limited capacity of STM with complex learning materials presented as a realistic training multimedia or multimedia program. However, this is not in accordance with Mayer’s (2005) *proximity* principle (Contiguity Principle) in multimedia learning, which is based on the integrated double code (ITC) hypothesis. On the contrary, the proximity principle claims that multi-presentation will give better LO than successive presentations.

However, this study also shows that the successive presentation of multimedia (Type II) was better than just text (analog) and speech supported with irrelevant images (Type I). This may indicate that a visual effect actually promotes LO when the representation forms are presented successively and there is good content relevance between the representation forms. In other words, this type of instrument does not seem to overload the STM capacity, and it is reasonable to assume that integrated double coding actually happens here in the STM preparation process. Thus, a successive instrument in a multimedia presentation facilitates information in such a way that the receiver is able to transform the different representation forms (image, voice, and text) into learning, where the different representation forms combine and complement each other (circuits between visual and verbal channels). This also corresponds with newer ITPC models on the integrated double coding process (Schnotz and Wagner, 2018). At the same time, this study reveals that ITPC processes also have their limitations in relation to actual LOs. In order for the integration to have a full effect on LOs, the given information should be presented successively, i.e., a little at a time in relation to visual and verbal information. The reason for this seems to lie in capacities related to the substructures in STM, especially substructures in the visual and verbal channels.

In other words, successive presentations help to facilitate integrated double coding without overloading the STM capacity. There was an increase in LO that corresponded with an increase in STM capability concerning this instrument. This may be interpreted to suggest that STM has a significant capacity to take advantage of this instrument, which also shows that much information can be simultaneously processed in an integrated double coding process when information is presented successively. There were significant differences in LOs at the *p* < 0.05 level between multimedia and text and Type II for both progressive visual and verbal capacities. These types of STMs measure show a capacity for increasing visual and verbal loads and therefore correspond with instrument characteristics in which information is presented in stages but constitutes a whole. This supports the assumption that it is the capacities of these two STM types that are essential to make or process information presented successively in a multimedia presentation.

A possible surprising result was that the version of the Ravens test (RAPM) of this study had such a clear effect on Type II in the regression analysis but not on the other two instruments in multimedia. There was a significant correlation between the LOs of Type II and Raven (*β* = 0.27, *p* < 0.001, *η*^2^ = 0.07). Raven measures a general non-verbal meta-cognitive ability, where the task is to find logical visual patterns in a progressive process. Here, the task was to find out the next step in a logical visual pattern progression from a given choice of eight options, with increasing difficulty from the first pattern to the last task. This puzzle solving involves putting together increasingly complex information. It is reasonable to assume that the process corresponds to cognitive skills, such as the STM capacity that is needed to process given information successively, which must be assembled into a whole to respond to knowledge questions and measure LO. However, in this study, we have not defined Raven measuring a specific STM capacity. Furthermore, it is reasonable to assume that increasing performance on Raven also assumes increasing STM capacity in general. Since Raven did not make a difference for the other two instruments, this might suggest that there may be a corresponding relationship between the ability to perform Raven and utilization of instrument Type II for learning. Indeed, Wiley et al. (2011) have also documented a high correlation between RAPM and individual differences in STM capacity.

Text was best compared with the instrument in multimedia that consisted of speech and irrelevant visual background material (Type I). Here, the learning material was presented with a visual commentary and background images without any special relevance heeded to the oral information. Former media research (Furnham et al., 1990) and cognitive research (Norman, 1969) have shown that voice alone is better than text, or that they both render equal LO. However, Mayer’s (2005) Multimedia Principle claims that words + pictures are better than words alone as information through both auditory-verbal (speech) and visual-verbal (text) channels. He refers primarily to speech (narrations), i.e., auditory-verbal presentation of words (Moreno and Mayer, 2002; Mayer et al., 2003). The Coherence Principle (Mayer, 2005) points out that learning occurs best when the presentation is free of irrelevant information. As instrument Type I contained background images without special relevance to the spoken material, the results are supported by Mayer’s Coherence Principle if we assume that the background images contributed to a Stroop effect and overloading of the STM capacity. However, there was no difference in LO between text and multimedia for instrument Type I for high STM capacity. For the individuals with low and medium STM capacity, learning proceeded best by text. This may indicate that the background images in Type I actually contributed to an STM overload for the individuals with low and medium capacity. For individuals with high capacity, the strain was not significant enough to cause any noticeable decrease in LO in relation to the text.

However, not all sub-types of the STM capacity were related to LO. PCvv had a particular connection to LO from multimedia through instrument Type I (*β* = 0.21, *p* < 0.01). It was also in this area that the differences in LO between the three capacity levels was greatest (*F* = 7.11, *p* < 0.001), with a clear increase in LO from low to high capacity. This may indicate that it was the capacity to process increasing visual-verbal information that had the greatest impact on LO from a multimedia presentation that was composed of speech and irrelevant images. It may also indicate that the progressive verbal capacity has helped in the mentioned focusing process, where being able to follow the voice-based information flow has had a greater impact on learning capacity than other capacity types. Furthermore, it was the individuals with low PCvv who had the lowest LO, even in relation to text. We did not measure the capacity for auditory-verbal capacity (PCav), but it is reasonable to assume that this would have been similar to the PCvv patterns in this material. Furthermore, PCvv was also the capacity category with the most similar characteristics compared with the tests used in this study; both measure the capacity for increasing verbal strain. One would, therefore, expect a similar relationship with the text presentation. Moreover, here, the difference in LO was significant between the capacity levels but not as marked (*F* = 3.96, *p* < 0.05). This may indicate that PCvv actually had a slight effect on the text presentation, but it did not mean less because this was a pure text, with no pictures or other irrelevant background information that would increase or burden the capacity load. The individuals could also read the text at their own pace, so it is likely that the flow of information did not overload the verbal STM capacity significantly, as the case is with multimedia, where the media controls the speed and density of information. It may, therefore, be reasonable to conclude that PCvv had a greater impact on LO with multimedia as a presentation composed of Type I instruments than pure text presentation. The individuals with high progressive visual-verbal capacity also learned the most from multimedia, although less in total compared to text.

Instrument Type III, which contained a multi-presentation with high mutual relevance, resulted in no difference in LO between multimedia and text. This may suggest that a multi-presentation no longer contributes to better LO compared with text, but neither does it inhibit LO. This may again indicate that multimedia and text with this instrument represent an approximately equal cognitive load, even if the structure is different. Mayer’s (2005) proximity principle is, therefore, unsupported by this study (see also the results from Type II). One possible explanation for this is that the recipients of multiple presentations selected information, and, in this case, specifically focused on the auditory-verbal information (speech). As the images were relevant to the speech, they did not contribute to impair LO through a Stroop effect, as in instrument Type I. There was a correlation between the progressive-verbal capacity and LO from the Type III instrument (*β* = 0.2, *p* < 0.01), and there was a significant difference in LO between low, medium, and high progressive verbal capacity (*F* = 4.21, *p* < 0.05). This may further indicate that this verbal capacity had an impact on how much auditory-verbal information could be processed together with the relevant and selected visual information. Unlike Type I, the Type III instrument was composed of relevant images and text in relation to speech, so the composition itself was designed to avoid a Stroop effect.

However, as for Type I, this result might also imply that attention was primarily focused on the verbal comments, and therefore the load for PCvv was of importance for the LO. Since the visual information was relevant to the speech, this may have helped in the preparation process between the channels (circuit output). Moreover, by doing so, this charged the visual-verbal capacity even more. This may, therefore, indicate that LO by methods that involve multi-presentation, in which voice is supported by relevant visual material, relies particularly on PCvv. This also provides support for the integrated double code hypothesis (Clark and Paivio, 1991), but not Mayer’s (2005) general multimedia principle. However, both the Contiguity Principle and Redundancy Principle are supported.

There were significant *p* < 0.05 level differences in LO between multimedia with instrument Type II and text for both visual and verbal channel capacities. The LOs were also larger than for text concerning the individuals with low, medium, and high visual and verbal channel capacity, but the relationship was the opposite for instrument Types I and III. This may indicate that a successive presentation does not overload the channel capacity and that this form of presentation involves an adapted CLS for both the visual and verbal channels. There was also a steady increase in LO between low, medium, and high channel capacity for the three instruments in multimedia and also for text.

This may indicate that individual differences in channel capacity are important in general for LO from multimedia and text, but the study did not show that individual differences in channel capacity had any effect on LOs from the three instruments in multimedia. The reason why Type II gave the best LO in the two channels can thus be explained by the substructures of the channels: PCvv for the verbal channel and PCvi for the visual channel.

## Conclusion and Implications

This study shows that successive multimedia presentation provides better LO compared with multi-presentation (synchronous), for all. However, the individuals with higher STM capacity learned relatively better than the individuals with lower STM capacity. In relation to the total CLS from instruments in multimedia-based presentations, successive presentations with the different representation forms (voice, text, and images) provide better LOs than both analogue text and other instruments in multimedia as multi-presentation, and speech with a visible commentator and irrelevant background images. There was no difference in LO between multi-presentation and pure text.

These CLT-oriented main results, yielded with the three tools in multimedia, can be expressed as follows by a CLS-LO formula, which was introduced as a theoretical analysis and pedagogical tool for expressing complex relationships between CLS and LOs:

$${\mathrm{CLS}}_{\mathrm{Type}\;\mathrm{II}\;I}>{\mathrm{CLS}}_{\mathrm{Type}\;I}>{\mathrm{CLS}}_{\mathrm{Type}\;\mathrm{II}}\to {\mathrm{LO}}_{\mathrm{Type}\;\mathrm{II}}>{\mathrm{LO}}_{\mathrm{Type}\;I}={\mathrm{LO}}_{\mathrm{Type}\;\mathrm{III}}$$

Theoretically, this study supports newer ITPC models. The channel capacity has general significance for LO but not for the utilization of the three instruments tested. Nevertheless, the study has shown that there is a need for a more nuanced understanding of the prerequisites that should be present for the integration between the visual and verbal channels to work well in relation to processing information for the best LO. An important prerequisite is that the information is provided successively, so that the interaction between the visual and verbal channels is not reduced because of capacity loads in and between the channels. The study also showed that it was certain substructures at the channel capacities that were most important for this. It was especially progressive (successive) visual and verbal channel capacities that had the greatest significance for LOs. These two structures are also an empirically grounded contribution to the theoretical development of a more nuanced channel architecture by the ITPC models.

As an overall conclusion, the results from this study are of importance for education, in general, as well as the design and composition of multimedia applications. The results can also affect adaptation to special education software, where people with reduced STM are likely to learn most from applications that are built with successive principles. However, in this context, it may be necessary to investigate which specific dysfunctions are in question in the STM. This study shows that there is capacity for increased visual and verbal workloads, which have a particular impact on LOs by successive presentations. However, even if instruments with low CLS are used in multimedia, the individuals with low capacity still learn relatively less than those with higher capacity. The finding related to the importance of substructures in the visual and verbal channels could also be a contribution to the further development of STM tests in special education or other purposes related to the mapping of cognitive abilities for children and adults.

This study primarily has a basic research perspective, but we also see several practical applications of the findings. The nuanced findings of this study on the relationship between presentation form, mental capacity, and learning will also be relevant within the field of crisis management as an analysis base for performance, crisis communication, decision-making, and shared situational awareness under high information pressure, noise, risk, and unpredictable conditions. For example, the finding of successive production of information could provide guidelines for composing orders and information sharing during crises, where information should then be given in “packages” with few words and possibly image sequences and then a short break of about 1–2 s before the next information is given. This must, of course, be included in training programs for crisis managers and operators, and adapted communication technology/apps can be developed for such CLT-based interaction. In addition, knowledge of the significance of specific substructures in the visual and verbal channels, as this research has shown, can be a contribution to the further development of tests and exercises in connection with the selection and competence development of personnel in key operational functions.

## Possible Limitations

It should be noted that all the respondents in this study were associated with higher education. For respondents with other backgrounds, the results of this study may be even more obvious considering the importance of STM capacity for learning by the three instruments.

A possible weakness of this study is the impact of the environment on the experiment, as the implementation occurred in classrooms under conditions similar to a normal teaching situation. This can reduce controllability. On the other hand, this was also the goal of the study: to investigate actual LOs and STM capacity under normal and daily learning conditions. Recent review studies by Choi et al. (2014) have shown that the environment has an impact on the measurement results and that there have been few effective studies under such conditions. Therefore, this study was also conducted under realistic conditions, as a contribution to this research.

There are nevertheless questions concerning which direction the environment affects the results, both individually and overall. At the individual level, the results regarding LOs and STM capacity could be lower than they would have been if the study had been conducted under controlled laboratory conditions. Noise, movements by other students, as well as other conditions in the classroom, may affect attention and concentration, thereby reducing performance. However, this will also apply to all respondents, and any bias will hardly occur in the same places found in the learning and performance process but rather in different places in the material of those respondents. This can affect the results of LOs on various questions in the instrument and at different places in the part tests of the STM test. However, since in this study we have merged the values and used average values as performance measures for both LOs and STM capacity, this will overall reduce the significance of the bias of an individual.

Thus, the results of this study should be able to provide a good picture of the importance of CLT capacity concerning LOs from multimedia with a realistic learning material, given under realistic surrounding conditions. In conclusion, it should also be mentioned that in this study we have not investigated the importance of the interactive use of multimedia, motivation, and emotional factors related to STM capacity, multimedia, and LOs. It is reasonable to believe that both the form of communication (of the curriculum) and the STM test (multimedia-based, vs. in paper form) can also have had an impact on the results. Recent studies where CLT is used as a theoretical basis (Leutner, 2014; Mayer and Estrella, 2014; Feldon et al., 2019) have also shown that motivational and emotional factors can have an impact on STM capacity and learning effects.

## Data Availability Statement

The datasets presented in this study can be found in online repositories. The names of the repository/repositories and accession number(s) can be found in the article/supplementary material.

## Ethics Statement

Ethical review and approval was not required for the study on human participants in accordance with the local legislation and institutional requirements. The patients/participants provided their written informed consent to participate in this study.

## Author Contributions

G-ET developed the theoretical basis and experimental design and completed the data collection. G-ET and OB wrote and revised the manuscript and approved the final version to be published. All authors contributed to the article and approved the submitted version.

### Conflict of Interest

The authors declare no potential conflicts of interest with respect to the research, authorship, and/or publication of this article. The views that are expressed in this article are solely those of the authors.
